# Dimensions of the ascending aorta in children and adolescents with repaired Tetralogy of Fallot obtained by cardiac magnetic resonance angiography

**DOI:** 10.1007/s00392-015-0912-6

**Published:** 2015-09-02

**Authors:** Matthias Grothoff, Meinhard Mende, Daniel Graefe, Ingo Daehnert, Martin Kostelka, Janine Hoffmann, Patrick Freyhardt, Lukas Lehmkuhl, Matthias Gutberlet, Anne Mahler

**Affiliations:** Department of Radiology, Heart Center, University of Leipzig, Struempellstr. 34, 04289 Leipzig, Germany; Clinical Trial Centre, University of Leipzig, Haertelstr. 16-18, 04107 Leipzig, Germany; Department of Pediatric Cardiology, Heart Center, University of Leipzig, Struempellstr. 34, 04289 Leipzig, Germany; Department of Cardiac Surgery, Heart Center, University of Leipzig, Struempellstr. 34, 04289 Leipzig, Germany; Department of Gynecology and Obstetrics, University of Leipzig, Liebigstr. 20a, 04103 Leipzig, Germany; Department of Radiology, Charité University Hospital, Augustenburger Platz 1, 13353 Berlin, Germany; Department of Intensive Care and Intermediate Care, University Hospital Aachen, Pauwelsstraße 30, 52074 Aachen, Germany

**Keywords:** Tetralogy of Fallot, Aortic aneurysm, Magnetic resonance angiography, Reference values

## Abstract

**Introduction:**

Dilatation of the ascending aorta is a common finding in Tetralogy of fallot (TOF). We sought to provide aortic dimensions in children and adolescents after corrected TOF obtained by contrast-enhanced cardiac-magnetic-resonance angiography (CE-CMRA) that could serve as reference values.

**Materials and methods:**

We enrolled 101 children and adolescents (56 male) with a median age of 10.9 years. All patients underwent CE-CMRA imaging using a 3-dimensional spoiled gradient-echo-sequence. Aortic diameters were measured at the level of the aortic valve (AV), aortic sinus (AS), sino-tubular junction (STJ) and the ascending aorta (AA) and compared with normal values obtained from literature. Sex-specific aortic dimensions are given as percentile curves as well as *z* scores. Furthermore CMR volumetric and functional parameters as well as clinical and anamnestic data were analyzed to identify parameters that are associated with aortic dilatation.

**Results:**

Diameters for aortic size for males were 3.6 + 16.6*BSA^0.5^ at the AV level, 7.0 + 19.5*BSA^0.5^ at the AS level, 7.0 + 14.4*BSA^0.5^ at the STJ level and 7.3 + 15.5*BSA^0.5^ at the AA level. Diameters for females were 5.8 + 14.1*BSA^0.5^ at the AV level, 7.2 + 17.6*BSA^0.5^ at the AS level, 5.2 + 15.4*BSA^0.5^ at the STJ level and 2.0 + 17.8*BSA^0.5^ at the AA level. All diameters in TOF patients were larger compared with normal values. The postoperative interval and age at examination were the only parameters associated with aortic size at all measured levels.

**Conclusion:**

We provide CE-CMRA data of aortic dimensions in children and adolescents after correction of TOF. Our data might be useful for an estimation of the “normal” aortic size in this patient cohort and can serve as a basis for future longitudinal studies adding prognostic data.

## Introduction

Tetralogy of Fallot (TOF) is the most common cyanotic congenital heart disease (CHD) [1]. Albeit TOF is defined by changes of the right ventricle and the pulmonary vessels, also dilatation of the aortic root and the ascending aorta can occur [2]. The pathomechanism of aortic dilatation in TOF and other cyanotic congenital heart disease associated with restricted pulmonary perfusion is unclear. The congenital anatomy, mostly prenatally manifested in form of right ventricular outflow obstruction or atresia and malalignment ventricular septal defect, which results in restricted flow to the pulmonary vessel and increased flow from both chamber to the aorta may play the major role. In such congenital heart disease with restricted pulmonary perfusion, the aortic dilatation is often detectable by fetal echocardiography. In addition, also genetic predisposition might contribute to aortic dilatation. It could, for instance, be shown that fibrillin-1 genetic mutations in TOF patients are associated with histologic abnormalities and larger aortic size [3–5].

Although rare, aortic dilatation can lead to the development of aneurysms and aortic dissection [6]. Therefore, early detection by close follow-up and timely intervention might help to improve the prognosis of fallot patients also with regard to aortic complications.

In literature, the prevalence of aortic root dilatation in TOF patients varies widely depending on the used imaging tool and the sizing criteria [2, 7]. Echocardiography is the first line imaging modality in CHD and previous studies have established reference values for aortic size after correction of TOF [8]. Besides echocardiography also cardiac-magnetic resonance (CMR) is a routine follow-up tool in these patients. Typical elements of CMR protocols are steady state-free precession (SSFP) sequences for the assessment of biventricular morphology, size and function. Recently, age- and sex-specific pediatric reference values for ventricular volumes have been published [9]. In many centers including ours, also contrast-enhanced CMR angiography (CE-CMRA) is part of the follow-up imaging protocol. It is a robust tool to visualize the morphology of the great vessels and offers the possibility of multiple measurements at different positions in multiplanar reconstructions during post processing.

The aim of this study was to report diameters of the aortic root and the ascending aorta (AA) obtained by CE-CMRA in children and young adults after correction of TOF and compare them to normal values derived from literature. We furthermore sought to identify parameters that are associated with aortic dilatation.

## Materials and methods

### Patient population

This study consists of 101 consecutive patients younger than 21 years of age after surgical correction of TOF undergoing CMR for follow-up. All patients came for regular outpatient visits to our tertiary care center. None of our patients was diagnosed with Marfan syndrome, Ehlers-Danlos syndrome or any other connective tissue disorder. At the time of the CMR examination none of our patients had a right-to-left shunt or a major aortopulmonary collateral artery.

### CMR image acquisition

All CMR examinations were performed on a 1.5 Tesla scanner (Gyroscan ACS-NT, Philips Healthcare, Best, The Netherlands) with a five-channel phased-array surface coil in supine position.

CE-CMRA images of the aorta were acquired using a 3-dimensional spoiled gradient-echo sequence in a coronary orientation with a repetition time of 2.9 ms, echo time 1 ms, flip angle 30° and matrix of 480 × 480 × 48. The field of view was adjusted to the patient size. Typical voxel size was 0.9 × 0.9 × 1.8 mm^3^, acquisition time 12 s. Two CE-CMRA examinations were performed in each patient. The first acquisition was manually started when the contrast appeared in the pulmonary trunc, the second acquisition was started automatically immediately after the first sequence for visualizing the aorta. In children younger than 6 years a weight adapted dose of 0.2 mmol/kg bodyweight Gadopentetat-Dimeglumin was administered. In children older than 6 years with a body weight <50 kg, 0.2 mmol/kg bodyweight Gadubutrol was administered, in all others a standard dose of 15 mmol Gadubutrol was used. Injection rate was 2 ml/s followed by a saline solution of 30 ml with the same injection rate.

For the assessment of biventricular volumes and masses, a breath hold or free breathing navigator Cine-steady-state-free-precession (SSFP) sequence with retrospective ECG-gating in an axial orientation and a slice thickness of 6 mm was used. Further sequence details have been published previously [10].

### CMR image analysis

**Fig. 1:**
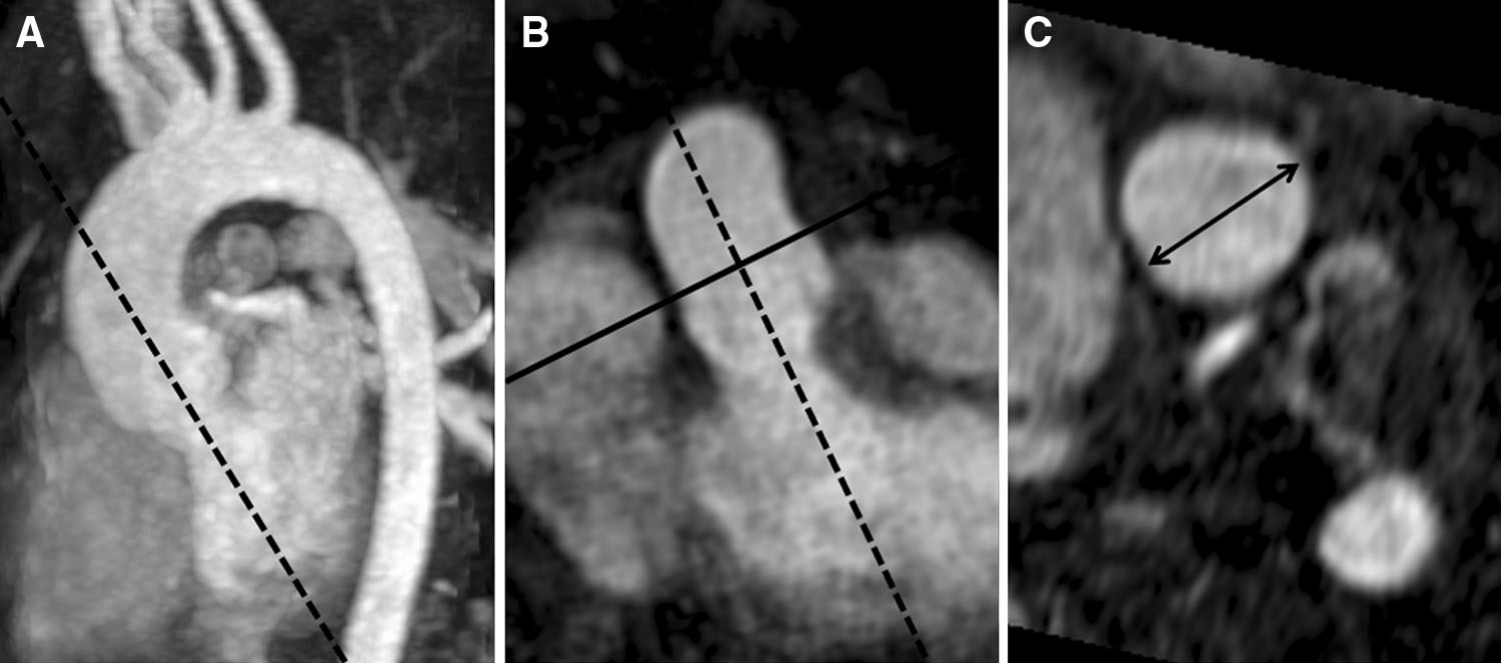
Measurement of aortic diameters. Maximum intensity projection (MIP) in a left anterior oblique orientation (**a**). Longitudinal MIP perpendicular to the first (**b**). Cross-sectional plane perpendicular to both longitudinal views at the level of the sino-tubular junction (**c**)

Measurements of aortic size were performed off-line on a separate workstation (Sectra PACS IDS5 11.4) by two blinded investigators with 4 (A.M.) and 15 (M.Gr.) years of experience in CMR. The aortic diameters were measured at four standardized levels, consisting of the aortic valve (AV), the aortic sinus (AS), the sino-tubular junction (STJ) and the AA. Each aortic segment was reconstructed in two double oblique planes as previously published [11]. First a parasagittal/paracoronal maximum intensity projection (MIP) of the aorta was reconstructed corresponding to a left anterior oblique view (Fig. 1). Then a second longitudinal plane perpendicular to the first plane was reconstructed. At last a third cross-sectional view perpendicular to both longitudinal views was angulated. At the level of the AS, the STJ and the AA the widest diameters were measured [12]. The aortic valve was measured at the level of the most basal attachment points of the three cusps to the aortic wall. Here two diameters perpendicular to each other were measured and the mean value was calculated.

Ventricular volumes were obtained by Simpson’s rule according to the protocol of the German Competence Network of Congenital heart disease [13]. All parameters were related to body surface area (BSA) and shown as indices. We determined left ventricular (LV) and right ventricular (RV) end-diastolic volume index (EDVI), end-systolic volume index (ESVI) and stroke-volume index (SVI). Furthermore, we calculated biventricular ventricular ejection fractions (EF).

### Anamnestic and clinical data

The parameters age at surgical correction of TOF, age at CMR examination, postoperative interval, type of correction and number of previous palliative procedures were taken from patient records. New York Heart Association (NYHA) functional class as determined by a standardized questionnaire, the degree of aortic insufficiency (AI) measured by echocardiography according to the guidelines of the European Society of Cardiology and non-invasive arterial blood pressure (given in percentiles) were obtained during the clinical routine follow-up examination at the day of the CMR examination [14].

### Comparison with normal values

Normative data were derived from the literature [11]. Aortic diameters could be compared at three corresponding levels (AS, STJ and AA). The equations for the percentiles of aortic diameters given in the cited study were used to calculate the predicted aortic diameters for the Fallot patients in the present study.

### Statistical analysis

For statistical analysis IBM SPSS Statistics software package 20.0 (Chicago, IL, USA) was used. Intra- and interrater coefficients of variation (CV) were estimated by means of ANOVA models with repeated measurements.

Checking the functional dependency, we found that aortic diameters fitted best to square root of BSA. Non-significant equivalence tests between girls and boys indicated that curves should be fitted separately for both sexes.

To calculate reference percentile curves the GAMLSS methodology [15] was used. It allows fitting several probability distributions with covariate dependent scale and shape parameters. Square root of BSA was chosen as appropriate covariate. Minimal global deviance as well as relatively smoothed curves and good agreement of estimated and observed quantiles were criteria for the choice of adequate model function. Skew exponential power family was applied four times, *t* family two and skew *t*3 family and Box-Cox *t* family one time each.

Aortic diameters of our TOF patients were compared with previously published normal values using paired *t* testing [11]. Furthermore our values were classified in a *z* value coordinate system basing on the previously published normal values.

Looking for covariates associated with aortic diameters, we proceeded in three steps in analogy to Sauerbrei and Schumacher [16]. First we performed bagging procedures based on 15 CMR volumetric and functional parameters as well as clinical and anamnestic parameters, i.e., each 100 linear models from bootstrap samples were fitted using the best subset criterion based on the Akaike Information Criterion. In step two we included only such variables that were in more than 50 % models into a linear model. In step three potentially a non-significant variable had to be excluded to get the final model.

## Results

The study cohort consisted of 56 male and 45 female patients with a median age of 10.9 years (range 10.1–20.2 years). The median BSA calculated from the Mosteller formula was 1.2 m^2^ (range 0.6–2.2 m^2^) with a median height of 133 cm (range 92–182 cm) and a median weight of 28.5 kg (range 12.0–94.8 kg) [17]. The median body mass index was 16.0 kg/m^2^ (range 11.8–32.7 kg/m^2^).

Parameters of the control cohort [11] were almost identical (Table 1).

**Table 1:** Patient parameters

	Fallot patients	Controls derived from [11]
Number of patients	101	53
Male:female (%)	55:45	57:43
Age (year)	10.9 (2–20)	9 (2-20)
Body surface area (m^2^)	1.02 (0.55–2.1)	1.05 (0.52–1.9)
Height (cm)	133 (92–182)	131 (81–184)
Weight (kg)	28.5 (12–95)	30 (12–75)

Continuous data are presented as median and range

**Fig. 2:**
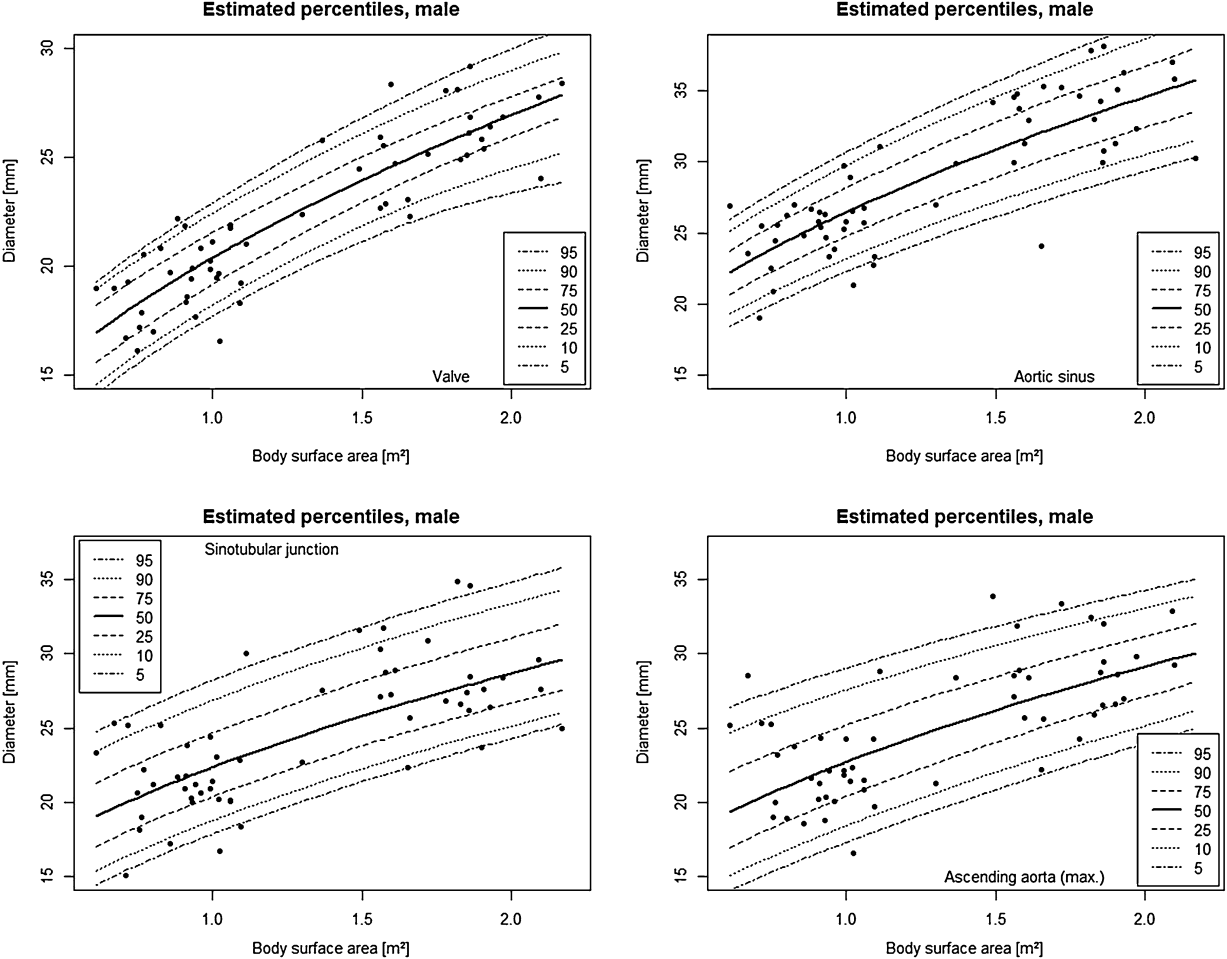
Percentile *curves* for aortic diameters after correction of TOF in male children and young adults

**Fig. 3:**
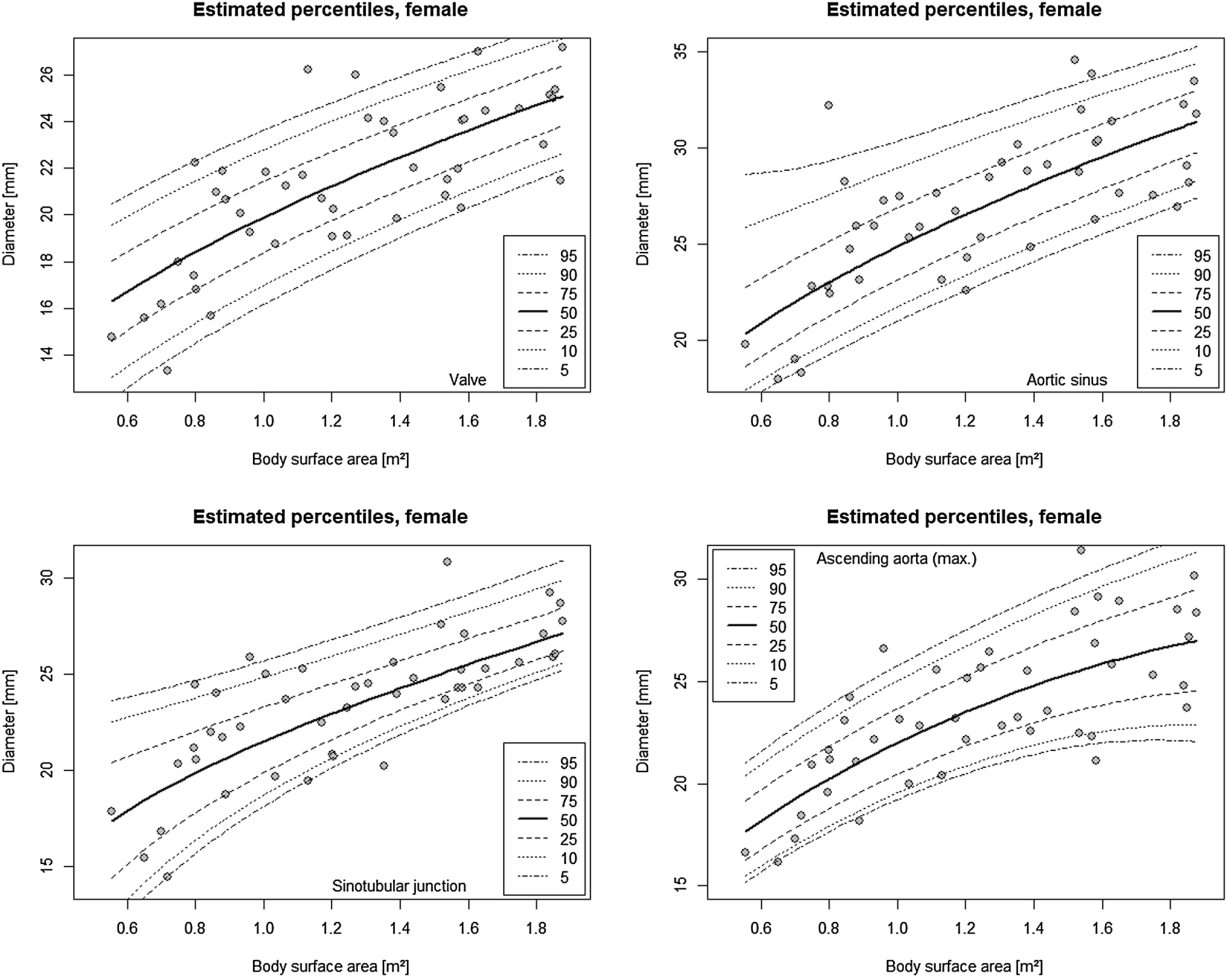
Percentile *curves* for aortic diameters after correction of TOF in female children and young adults

The median and range of the measured aortic segments are given in Table 2. Mann–Whitney *U* test of the indexed aortic diameters revealed no difference between male and female patients. However, because equivalence testing procedure failed to show equivalence, sex-specific values are given. Percentile equations are shown in Table 3. Percentile curves are shown in Figs. 2 and 3.

**Table 2:** Sex-specific diameters of the aorta

	Males		Females	
	Median	Range	Median	Range
Aortic valve (mm/m^2^)	18.3	11.4–31.2	17.7	11.5–27.9
Aortic sinus (mm/m^2^)	22.6	14.0–44.1	22.3	14.8–40.3
Sino-tubular junction (mm/m^2^)	19.9	11.5–38.2	18.7	14.0–32.2
Ascending aorta (mm/m^2^)	20.3	10.4–42.5	19.3	12.8–30.0

**Table 3:** Sex-specific functions for aortic diameters

	Males		Females	
	Predicted diameter	SD of residuals (mm)	Predicted diameter	SD of residuals (mm)
Aortic valve	3.6 + 16.6*BSA^0.5^	2.4	5.8 + 14.1*BSA^0.5^	2.3
Aortic sinus	7.0 + 19.5*BSA^0.5^	3.5	7.2 + 17.6*BSA^0.5^	3.2
Sino-tubular junction	7.0 + 14.4*BSA^0.5^	4.3	5.2 + 15.4*BSA^0.5^	3.5
Ascending aorta	7.3 + 15.5*BSA^0.5^	4.0	2.0 + 17.8*BSA^0.5^	3.5

**Fig. 4:**
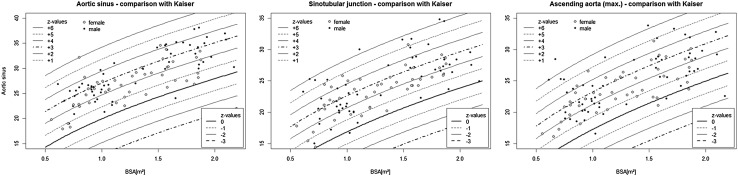
*z* values of aortic diameters in TOF compared with normal aortic values according to [11]

Compared with normal values the Fallot group showed larger aortic diameters at all comparable levels with significant differences between the predicted [11] and measured diameters (Fig. 4). The mean differences and standard deviations were: 5.9 ± 2.9 mm at the AS level, 5.2 ± 2.9 mm at the STJ level and 4.8 ± 3.1 mm at the AA level (*p* < 0.001 for all).

For readers convenience we provide online electronic calculators of *z* scores when compared with the Fallot group (http://tof.pedz.de) and when compared to normal aortas (http://mri.pedz.de).

The coefficients of variations for intraobserver variability were: 8 % at the AA level, 6.3 % at the AS level, 5.8 % at the STJ level and 5.9 % at the AA level. The coefficients of variations for interobserver variability were: 6.5 % at the AA level, 6.1 % at the AS level, 6.0 % at the STJ level and 5.8 % at the AA level.

In CMR planimetry median LV-EDVI was 79.6 ml/m^2^ (range 40–172 ml/m^2^), LV-ESVI was 34.5 ml/m^2^ (range 15–92 ml/m^2^), LV-SVI was 46.0 ml/m^2^ (range 21–120 ml/m^2^) and LV-EF was 56.0 % (range 28–79 %). Median RV-EDVI was 110.6 ml/m^2^ (range 53–215 ml/m^2^, RV-ESVI was 57.0 ml/m^2^ (range 18–173 ml/m^2^), RV-SVI was 53.1 ml/m^2^ (20–176 ml/m^2^) and RV-EF was 47.9 % (range 25–74 ml/m^2^).

No significant associations were found between these parameters and aortic dimensions at the four measured levels.

Median age at surgical correction was 0.8 years (range 0.1–11.9 years), median postoperative interval was 9.7 years (range 2.0–19.1 years). A transannular approach was used in 74/101 patients, a subvalvular patching in 18/101 patients, a conduit was used in 7/101 patients. In two patients data regarding the used surgical method were inconclusive or missing. In 16/101 patients palliative procedures had been performed, 14 patients had one palliation and one patient had two and three palliations, respectively. Pulmonary atresia was corrected in 6/101 patients.

The postoperative interval and age at CMR examination were the only parameters that were associated with aortic size at the four measured levels (*p* < 0.001 for all), meaning patients with a longer interval and a higher age had larger aortic dimensions. These two parameters were highly interdependent as the majority of patients were corrected within the first year of life. Age at corrective surgery, type of correction as well as palliations were not associated with aortic size. Also the presence of a right aortic arch, which was found in 25 patients (25 %), and pulmonary atresia were not associated with aortic size.

NYHA functional class I was found in 74/101 patients, class II in 18/101 patients and class III in 1/101 patients. In eight patients data were missing. NYHA functional class was not associated with aortic size.

Echocardiography showed competent aortic valves in 75/101 patients, a mild AI in 20/101 patients and a moderate AI in 2/101 patients. In four patients data were inconclusive or missing. Median systolic blood pressure was below the 90th percentile, median diastolic blood pressure was on the 50th percentile. AI and blood pressure were not associated with aortic size.

## Discussion

It is well-known that TOF can be associated with a clinically relevant dilatation of the aorta and echocardiography is the first line imaging tool for measuring aortic dimensions. In addition to echocardiography also CMR is an inherent part of the follow-up work flow in congenital heart disease and recently a number of CMR derived normative date have been published [18–21].

Our study adds CMR data of the aortic size in children and adolescents after correction of TOF that could serve as reference values.

Besides the unique capabilities of CMR in imaging morphologic and functional changes of the right heart after correction of TOF also imaging of the great vessels is feasible without restrictions from patient constitution and the presence of an adequate acoustic window. In most centers the CMR protocol includes the administration of a gadolinium based contrast-medium for CE-CMRA and late gadolinium enhancement (LGE) imaging. The CE-CMRA sequence is primarily used for visualization of the right ventricular outflow tract and the pulmonary arteries. Yet the short acquisition time also allows imaging of the entire thoracic aorta with optimal timing using the same contrast bolus.

The acquisition of a 3-dimensional CE-CMRA dataset offers some advantages over other CMR techniques like 2-dimensional spin-echo, phase contrast or steady state-free precession imaging. In a 3-dimensional CE-CMRA dataset it is possible to create multiplanar reconstructions and to perform multiple measurements. Once the source data is acquired all measurements can be performed off-line in any position and there is no need for a correct cross-sectional angulation pre imaging. In our study we provide normal values in four positions of the aortic root and the ascending aorta. As known from echocardiography studies, enlargement in postoperative TOF is not limited to a single section of the aorta but can effect multiple segments [22, 23]. Thus a gapless acquisition might be better than the acquisition of single slices. Moreover CE-CMRA sequences are based on differences in *T*1 relaxation times and not on inflow effects what gives them a high signal to noise ratio and makes them insensitive to flow artifacts.

The robustness of this method is underlined by a low intra- and interobserver variability of our data. The reliability of measurements is particularly important in CHD as therapeutic decisions are often based on the time course of morphological and functional changes. Therefore the capability to detect minor changes and tendencies is crucial. Interobserver variability was highest at the level of the AV and the AS and lowest at the level of the AA. This is a common finding in CE-CMRA measurements of the aorta as the 3-dimensional dataset is acquired over multiple cardiac cycles without electrocardiographic triggering and irrespective of vessel wall motion. Therefore the contours of the highly pulsatile proximal vessel sections show more blurring than the more distal sections [11, 24].

Although growth of cardiovascular structures is an allometric process, indexing cardiac parameters to BSA is common practice not only in adult but also in pediatric cardiology [25]. According to previous studies our data show that growth of the aorta is best described by a linear relationship between aortic diameters and the square root of the BSA [11, 26]. In addition we provide sex-specific values. Previous studies found bigger valve diameters in boys at all ages, even before adolescence [27, 28]. Moreover onset and time course of growth is sex- specific. Pooled values would have underestimated male and overestimated female aortic diameters.

Aortic diameters of our TOF cohort are significantly larger than the normal aortic values published by Kaiser et al. [11]. Numerous previous echocardiography studies have reported enlarged aortas in TOF, yet the prevalence of aortic dilatation varies widely depending on definition and cut-off values [2, 7, 29]. In a large multicenter study an aortic root diameter of ≥40 mm was found in 29 % of adult TOF patients [22]. However, there is only little data on aortic size measured with CMR. Recently Kutty et al. published CMR data of AA size in a large group of TOF patients [18]. Compared with our data AA diameters were slightly higher in the cited study, which might be explained by methodological differences in AA assessment. Kutty et al. used phase contrast imaging for measuring the AA, meaning that the aortic wall was included into the diameters. In CE-CMRA the lumen of the aorta appears hyperintense caused by the gadolinium based shortening of *T*1 relaxation times. The surrounding tissue including the vessel walls is hypointense. Measurements in CE-CMRA therefore exclude vessel walls and can rather be compared to luminography in conventional angiography. Moreover age at repair was higher in the cited study. The majority of our patients underwent corrective surgery within the first year of life and might therefore be considered a more ‘contemporary’ TOF cohort of children and adolescents as an early repair is standard in most centers nowadays.

Yet it is unclear whether an early repair can reduce, delay or even prevent aortic dilatation in TOF. Though it is assumed that increased aortic flow attributable to right-to-left shunting might lead to high wall shear stress also intrinsic alterations of the aortic wall seem to play a role in the pathophysiology [30]. Actually our data did not show a significant association between the age at repair and aortic size. The only parameters associated with an increased diameter at all measured levels were the postoperative interval and the age at CMR examination. As our group was quite homogeneous with regard to an early repair, these two parameters were highly interdependent and should be interpreted as a continuous growth of aortic dimensions over time. Our results support the thesis of an intrinsic aortopathy rather than the thesis of preoperative hemodynamic alterations.

Male sex was associated with non-significant larger aortas. In contradiction to previous studies neither right aortic arches nor increased LV volumes were associated with aortic dilatation [2, 4]. Large prospective longitudinal multicenter studies are necessary to clearly identify independent factors of aortic dilatation in TOF.

The natural course of the left-sided affection of TOF is discussed controversially [23, 31]. The mild degree of aortic dilation in repaired TOF patients as shown by our results reflects our clinical experience. Severe complications due to this dilation are rare and there only exist sporadic reports of aortic dissection or even rupture. Yet in future decades the number of adult and elderly patients with corrected TOF will increase and atherosclerosis may put these dilated aortas at risk for dissection. In addition a dilated aorta could result in a compression of the right pulmonary artery with unequal pulmonary perfusion and in the worst case leading to left-sided pulmonary hypertension with the need of a proximal aortic replacement [32]. Moreover, aortic dilatation at the level of the AV and the AS can lead to AI. In the present study AI was obtained by echocardiography and there were only a small number of young patients showing mild to moderate AI. This might be the reason for the absence of a correlation between aortic size and aortic regurgitation. Further studies should use CMR phase contrast flow measurement for an absolute quantification of AI and a more detailed analysis.

Our results show the distribution of aortic dimensions in patients with corrected TOF. This data may be useful for an estimation of the ‘normal’ aortic size in this patient cohort.

Special guidelines regarding the optimal time of replacement of the ascending aorta in patients with connective tissue diseases (e.g. Marfan syndrome) as well as normal variants (e.g. bicuspid aortic valve) have been published [12]. No such cut-off values exist for patients with corrected TOF. Although we cannot provide such data with our current approach this study might be a basis for future longitudinal studies using CE-CMRA.

## Limitations

The smallest BSA in this study was 0.52 m^2^. Therefore, we cannot provide reference values for neonates and very young infants. However, this group of patients is not anyway routinely examined by CMR in follow-up examinations in TOF as the procedure is time consuming and needs sedation.

Although our study population consisted of a relatively large number of TOF patients, it is a cross-sectional study with the given limitations in identifying parameters that are associated with aortic growth. Larger longitudinal studies might give further insight into the time course and risk factors of aortic dilatation in TOF.

At last this study has the typical limitations of a retrospective approach. Some data regarding the type of surgery and palliative procedures were missing in our analysis.
